# Echocardiographic Findings in Saudi Adults With Type 2 Diabetes Mellitus in the Jazan Region

**DOI:** 10.7759/cureus.112673

**Published:** 2026-07-14

**Authors:** Mohammed I Sha Aldeen, Asma Alamin, Meaad Elbashir, Moawia Gameraddin, Babiker A Awadalla Awadelseed, Mohammed O Khider, Hanan Elnour, Sara Ali, Elrashed AbdElrahim, Awadia Gareeballah

**Affiliations:** 1 College of Graduate Studies, Sudan University of Science and Technology (SUST), Khartoum, SDN; 2 Department of Radiology, College of Applied Medical Sciences, University of Hail, Hail, SAU; 3 Department of Diagnostic Radiography Technology, College of Nursing and Health Sciences, Jazan University, Jazan, SAU; 4 Department of Diagnostic Radiology, College of Applied Medical Sciences, Taibah University, Medina, SAU; 5 Department of Radiological Sciences and Medical Imaging, Faculty of Applied Medical Sciences, Northern Border University, Arar, SAU; 6 Department of Diagnostic Radiology, Karary University, Khartoum, SDN; 7 Department of Diagnostic Radiology, Taif University, Taif, SAU

**Keywords:** cardiac abnormalities, cardiovascular risk, diabetes duration, echocardiography, type 2 diabetes mellitus

## Abstract

Background and aim

Type 2 diabetes mellitus (T2DM) is associated with structural changes in the heart and an increased risk of cardiovascular disease. Despite being a common health issue in Saudi Arabia, studies focusing on echocardiographic findings among patients with diabetes in the Jazan region are limited. The aim of this study was to determine the prevalence and patterns of echocardiographic abnormalities in adult patients with T2DM and identify the demographic and clinical risk factors associated with these abnormalities in the Jazan region.

Methods

This retrospective study included 168 adult patients with T2DM from two tertiary hospitals in Jazan, Saudi Arabia, who underwent transthoracic echocardiography. Demographic details and clinical history, including BMI, hemoglobin A1c (HbA1c), and diabetes duration, were retrieved from patients’ electronic medical records. Statistical analyses, including chi-square tests and logistic regression, were used to determine the relationship between patient characteristics and echocardiographic findings.

Results

Overall, 85.12% of patients had cardiac abnormalities, with mitral regurgitation (40.5%), left ventricular hypertrophy (30.4%), and tricuspid regurgitation (27.4%) being the most prevalent findings. The prevalence was higher among male patients and those aged 41-60 years and >60 years. A diabetes duration of ≥10 years and poor glycemic control (HbA1c ≥7%) were associated with higher odds of cardiac abnormalities.

Conclusions

In Jazan, echocardiographic abnormalities are common among patients with T2DM. Although diabetes duration of ≥10 years and poor glycemic control (HbA1c ≥7%) showed significant crude associations, both remained independently associated with echocardiographic abnormalities in the multivariate model. Routine cardiac screening should be considered, regardless of age, sex, or BMI, as it may help identify and manage cardiac abnormalities.

## Introduction

Diabetes mellitus (DM) is among the most common chronic metabolic disorders, and its prevalence has significantly increased worldwide in recent decades. According to the World Health Organization, Saudi Arabia has the second-highest rate of diabetes in the Middle East and the seventh-highest rate worldwide, with a prevalence of 25.4% among individuals aged ≥30 years [1]. Age plays a significant role in the increased prevalence of DM, reaching 40.2% among patients aged ≥45 years, who are therefore susceptible to developing diabetes-related complications that greatly increase the risk of morbidity and mortality. Cardiovascular disease (CVD) is the primary cause of mortality among individuals with diabetes [2-4].

Diabetes is associated with cardiac alterations, including myocardial thickening and primarily diastolic dysfunction, collectively known as diabetic cardiomyopathy. Multiple previous studies have reported a significant prevalence of diastolic dysfunction among patients with diabetes [5,6]. In addition, due to advancing age and comorbidities such as hypertension and obesity, patients with type 2 DM (T2DM) are susceptible to other cardiac abnormalities that affect prognosis, including left ventricular hypertrophy (LVH), reduced left and right ventricular ejection fractions, left atrial dilation, and valvular disorders [7-10].

Echocardiography is an important tool for diagnosing cardiomyopathy. It is considered the gold standard for assessing cardiac structure and function [11,12]. It is also useful for early diagnosis and timely management. Furthermore, echocardiography is a safe and noninvasive imaging modality.

T2DM has reached epidemic proportions worldwide, particularly in Saudi Arabia [1]. While much has been done to raise awareness of diabetes and its complications, many individuals remain undiagnosed and are therefore at risk of serious health complications, including CVD. Among the many complications of diabetes, CVD is the most concerning, and many patients with diabetes develop both structural and functional cardiac abnormalities. Clinical consequences of cardiac alterations, including diastolic dysfunction, LVH, and valvular disorders, are frequently observed in patients with diabetes. Therefore, early detection is essential. Echocardiography is one of the most important noninvasive and accessible imaging technologies for detecting these abnormalities. However, few studies in the Gulf region have examined the prevalence of echocardiographic abnormalities in patients with T2DM. This gap highlights the need for further studies in the region and emphasizes their importance for local clinical practice and public health.

The main objective of the present study is to assess the frequency and patterns of echocardiographic findings in adult patients with T2DM in Jazan, one of the southern regions of Saudi Arabia. The study aims to provide a better understanding of how different cardiac abnormalities identified on echocardiography in patients with T2DM correlate with demographic and clinical parameters, such as age, sex, and BMI, as well as how these parameters influence hemoglobin A1c (HbA1c) levels and diabetes duration. The study aims to elucidate the degree of cardiac risk faced by patients with diabetes and provide a foundation for identifying cardiac abnormalities, which may help guide management strategies to improve patient outcomes and healthcare delivery in the region.

## Materials and methods

Study design

This retrospective study included 168 patients with T2DM from King Fahad Central Hospital and Prince Mohammed bin Nasser Hospital, Jazan, who underwent echocardiographic evaluation due to signs and symptoms of cardiac disease. Approval for this study was obtained from the Jazan Health Cluster Research Ethics Committee (approval number: 2462). Patients were selected using a systematic random sampling technique. Data were extracted from electronic medical records (EMRs) using a structured clinical data sheet that included demographic information, including sex, age, and BMI. In addition, diabetes duration, clinical history, and HbA1c levels were collected.

Data collection process

Data were collected through a systematic process involving extraction from EMRs using a specifically designed structured clinical data sheet to collect information relevant to the study objectives. The data collection process included defining data elements, developing the structured clinical data sheet, accessing EMRs, extracting data, ensuring quality assurance, storing data, and validating data.

Inclusion criteria

The evaluated sample included male and female participants aged 14-90 years with varying durations of T2DM diagnosed according to American Diabetes Association guidelines who underwent echocardiographic evaluation between 2022 and 2025.

Exclusion criteria

Several clinical and pathological conditions were excluded, including type 1 DM, T2DM complicated by end-stage renal disease, rheumatic and valvular heart disease, pregnancy, and urinary tract infections or any other conditions causing proteinuria. Furthermore, patients with congenital cardiac diseases were excluded.

Echocardiographic evaluations

Transthoracic echocardiographic imaging was performed by cardiologists using a Philips EPIQ 5C Ultrasound System (Philips, Amsterdam, the Netherlands). Patients were placed in the supine position and turned to the left lateral decubitus position with the arm elevated and resting under the head. A low-frequency transducer was used (2.5-3.5 MHz). Parasternal long-axis, parasternal short-axis, and apical window (PA2CH and PA4CH (two- and four-chamber)) views were obtained. A final diagnosis was established by a certified echocardiography specialist using the diagnostic criteria set by the American Society of Echocardiography and the European Association of Cardiovascular Imaging.

Statistical analysis

Data analysis was performed using IBM SPSS Statistics for Windows, version 27.0 (released 2019; IBM Corp., Armonk, NY, USA). Descriptive statistics are presented as frequencies and percentages. A chi-square test was used to assess the association between demographic characteristics and diabetes duration with the presence of cardiac abnormalities. In addition, logistic regression analysis, including univariate (crude OR) and multivariate (adjusted OR) analyses, was performed to estimate the ORs for the presence of disease-related conditions after adjusting for demographic and clinical factors, including age, sex, BMI, HbA1c, and diabetes duration. A p-value <0.05 was considered statistically significant.

## Results

The results delineate the demographic and clinical characteristics of adult patients with T2DM, followed by the echocardiographic findings identified in the study cohort. The occurrence and characteristics of cardiac abnormalities are described, along with their distribution according to age, sex, BMI, glycemic control (HbA1c), and diabetes duration. Additionally, univariate and multivariate logistic regression analyses were conducted to identify independent factors associated with echocardiographic abnormalities.

The study found that the most represented age groups among patients with T2DM were 41-60 years and >60 years, comprising 81 (48.2%) and 49 (29.2%) patients, respectively. More than half of the patients were male (93, 55.4%), and the majority had an HbA1c level ≥7% (157, 93.5%). Most patients had a diabetes duration of 3-10 years and 11-20 years (72 (2.9%) and 67 (39.9%), respectively), and more than half had a normal BMI (88, 52.4%), as shown in Table 1.

**Table 1:** Demographic characteristics of patients with T2DM. All variables in the table are presented as frequencies (N) and percentages. Frequency (N) represents the number of participants within each category, while percentage (%) represents the proportion of participants in each category relative to the total study sample (N = 168). HbA1c, hemoglobin A1c; T2DM, type 2 diabetes mellitus

Characteristic	Category	N (%)
Age (years)	14-24	12 (7.1%)
	25-40	26 (15.5%)
	41-60	81 (48.2%)
	>60	49 (29.2%)
Gender	Female	75 (44.6%)
	Male	93 (55.4%)
HbA1c	<7% (controlled)	11 (6.5%)
	≥7% (uncontrolled)	157 (93.5%)
Diabetes duration (years)	3-10	72 (42.9%)
	11-20	67 (39.9%)
	>20	29 (17.3%)
BMI	18-25	88 (52.4%)
	>25	80 (47.6%)
Total		168 (100.0%)

**Figure 1:**
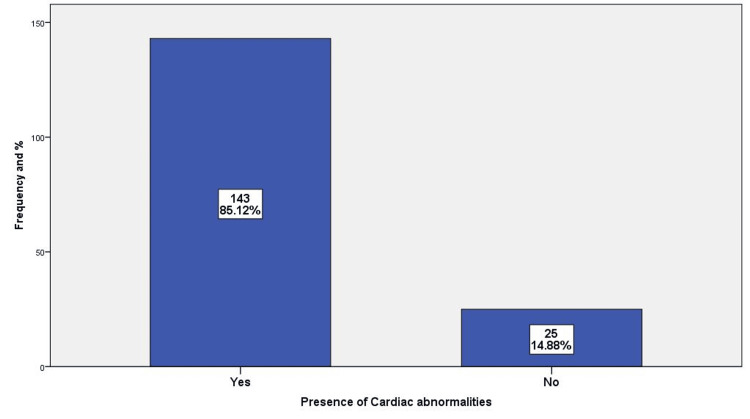
Prevalence of cardiac abnormalities in the study sample. The frequency and percentage represent the presence of cardiac abnormalities.

**Figure 2:**
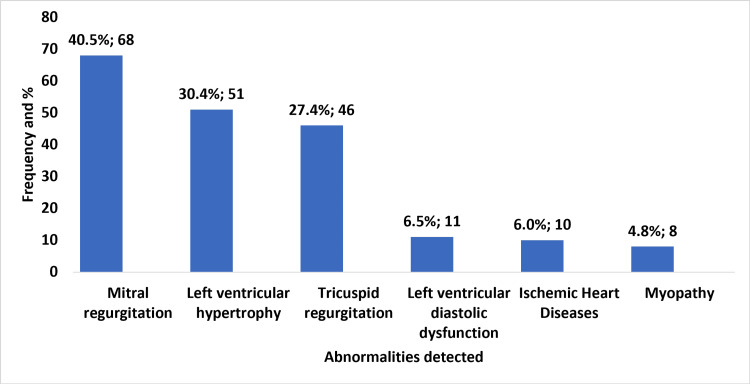
Echocardiographic findings detected in patients with T2DM. Distribution of echocardiographic abnormalities among patients with T2DM. The bars represent the number and frequency of each abnormality. The percentages were calculated using a total sample size of 168 patients. T2DM, type 2 diabetes mellitus

Figure 1 shows the overall prevalence of cardiac abnormalities detected by echocardiography among patients with T2DM. Cardiac abnormalities were identified in 143 (85.12%) of the study participants, indicating a high burden of echocardiographic abnormalities in this population. The types and distribution of specific echocardiographic abnormalities are shown in Figure 2. The most common finding was mitral regurgitation (68 patients, 40.5%), followed by LVH (51 patients, 30.4%) and tricuspid regurgitation (46 patients, 27.4%). Less frequent abnormalities included left ventricular diastolic dysfunction (11 patients, 6.5%), ischemic heart disease (10 patients, 6.0%), and cardiomyopathy (eight patients, 4.8%).

Table 2 presents the univariate and multivariate logistic regression analyses assessing the association between demographic and clinical factors and the presence of echocardiographic abnormalities in patients with T2DM. In the univariate analysis, age <60 years (crude OR = 3.869), HbA1c ≥7% (crude OR = 1.295), and diabetes duration ≥10 years (crude OR = 8.6) were associated with higher odds of developing cardiac abnormalities. Among these factors, only longer diabetes duration (≥10 years) showed a statistically significant association (p = 0.041). Additionally, BMI <25 kg/m² demonstrated significantly lower crude odds of the outcome (COR = 0.298; 95% CI: 0.117-0.757; p = 0.011). This association remained significant in the multivariate analysis (adjusted OR = 0.326; 95% CI: 0.125-0.849; p = 0.022).

**Table 2:** Univariate and multivariate logistic regression analyses of independent factors associated with echocardiographic findings in T2DM. This table shows the results of logistic regression analyses. Univariate logistic regression was used to estimate crude ORs, and multivariate logistic regression was used to estimate adjusted ORs for factors associated with the outcome. Each estimated OR and crude OR is presented with its corresponding 95% CI, and p-values <0.05 were considered statistically significant. HbA1c, hemoglobin A1c; T2DM, type 2 diabetes mellitus

Variables	Crude OR	p-value	95% CI	Adjusted OR	p-value	95% CI
Gender: Female	0.97	0.944	0.412-2.282	1.021	0.964	0.405-2.576
Gender: Male	0.969	0.944	0.404-2.277	0.995	0.991	0.395-2.503
Age <60 years	3.869	0.076	0.869-17.227	1.437	0.694	0.237-8.726
HbA1c ≥7%	1.295	0.751	0.263-6.379	1.106	0.907	0.206-5.932
Diabetes duration ≥10 years	8.6	0.041	1.095-68.413	5.968	0.152	0.517-68.833
BMI <25 kg/cm²	0.298	0.011	0.117-0.757	0.326	0.022	0.125-0.849

In the multivariate analysis, female gender (adjusted OR = 1.021), age <60 years (adjusted OR = 1.437), and duration of diabetes ≥10 years (adjusted OR = 5.968) showed increased odds of cardiac abnormalities, although these associations did not reach statistical significance (p > 0.05).

Table 3 summarizes the cross-tabulation of demographic variables, glycemic control, diabetes duration, and the presence of echocardiographic abnormalities. There was no statistically significant association between age and cardiac abnormalities (χ² = 3.59, p = 0.58), nor between gender and the presence of abnormalities (χ² = 0.00, p = 0.944). Similarly, HbA1c level (<7 vs. ≥7) was not significantly associated with echocardiographic findings (χ² = 0.10, p = 0.75). In contrast, a statistically significant association was found between BMI and cardiac abnormalities (χ² = 7.00, p = 0.008), with patients having a BMI of 18-25 kg/m² showing a higher proportion of abnormalities (81/88) compared with those with a BMI >25 kg/m² (62/80), with a small effect size (0.20). Additionally, diabetes duration showed a significant relationship with cardiac abnormalities (χ² = 8.36, p = 0.015), with prevalence increasing as disease duration increased: 76.4% in patients with 3-10 years of diabetes, 89.5% in those with 11-20 years, and 96.6% in those with >20 years of diabetes, indicating a progressive increase in cardiac involvement over time (effect size = 0.22).

**Table 3:** Cross-tabulation between demographic factors, diabetes duration, and the presence of echocardiographic abnormalities. This table shows the frequency distribution of the presence of abnormalities among different demographic groups, along with the corresponding chi-square values, p-values, and effect sizes used to assess group differences. A p-value <0.05 was considered statistically significant. * p < 0.05 ** p < 0.01 HbA1c, hemoglobin A1c

Demographic factors		Presence of abnormality		Total	χ²	p-value	Effect size
		No	Yes				
Age	<65	23	107	130	3.59	0.58	0.15
	>65	2	36	38			
Gender	Female	11	64	75	0.00	0.944	0.01
	Male	14	79	93			
BMI/kg/cm²	18-25	7	81	88	7.00	0.008**	0.20
	More than 25	18	62	80			
HbA1c	<7 (controlled)	2	9	11	0.10	0.75	0.020
	≥7 (uncontrolled)	23	134	157			
Diabetes duration (years)	3-10	17	55	72	8.36	0.015*	0.22
	11-20	7	60	67			
	More than 20	1	28	29			
Total		25	143	168	-		

## Discussion

This study provides significant insights into the echocardiographic characteristics of patients with T2DM in the Jazan region, highlighting the high incidence of cardiac abnormalities in this population. The results emphasize the importance of regular echocardiographic screening in patients with diabetes to detect cardiac abnormalities. Most patients had structural and functional cardiac changes, with mitral regurgitation, LVH, and tricuspid regurgitation being the most common findings. These findings add to the literature by demonstrating how diabetes duration and other clinical characteristics affect cardiac health and reinforce the importance of early detection and intervention to reduce the risk of adverse outcomes in this high-risk group.

The present study revealed that echocardiographic abnormalities were more prevalent in male than in female patients, particularly among those aged 41-60 years and >60 years. This finding is consistent with Al-Esawi and Amer, who identified advanced age, specifically >60 years, as a notable risk factor for diabetic complications [13]. Similarly, Koster et al. reported that men have a greater likelihood than women of developing coronary artery disease [14], whereas Ahmadi et al. found that both major and minor ECG abnormalities are more common in males and tend to increase with advancing age [15]. These findings underscore the significance of male sex and advanced age as potential predictors of cardiac disease in patients with T2DM, as assessed by transthoracic echocardiography.

The current study identified cardiac abnormalities in 85.12% of participants, indicating a high burden of echocardiographic abnormalities in this population. The most common abnormality was mitral regurgitation (40.5%), followed by LVH (30.4%) and tricuspid regurgitation (27.4%). Mitral regurgitation was not the most prevalent finding reported in previous studies. Zhao et al. reported higher left ventricular mass in patients with T2DM [16]. Additionally, Jørgensen reported that the prevalence of diastolic dysfunction, LVH, and left atrial enlargement was 19.4%, 21.0%, and 19.6%, respectively [17]. Similarly, several studies have reported findings consistent with our observation that LVH is the second most common echocardiographic abnormality [18-20].

In the univariate analysis, age <60 years, HbA1c ≥7, and diabetes duration ≥10 years were associated with higher odds of developing cardiac abnormalities. Consistently, Yao et al. reported that age and diabetes duration were associated with the risk of CVD [21]. Furthermore, Wan et al. reported positive log-linear correlations between HbA1c variability and CVD and mortality across all age groups. The hazard ratios for composite CVD and all-cause mortality indicated that age was inversely correlated with HbA1c variability [22]. Age is a crucial risk factor for CVD, and several cardiovascular risk prediction models include age as an independent risk factor for CVD. Evidence suggests that effective management of cardiovascular risk factors improves clinical outcomes, even among elderly patients [23]. These findings underscore the importance of early detection and proactive management of modifiable risk factors, including glycemic control and diabetes duration, to reduce the incidence of cardiac complications. Moreover, regular cardiovascular screening and personalized risk assessment, particularly for patients with prolonged diabetes or inadequate glycemic control, are crucial measures to improve long-term outcomes in this population.

The study found a significant association between BMI and cardiac abnormalities (χ² = 7.00, p = 0.008), with patients having a BMI of 18-25 kg/m² showing a higher incidence of abnormalities. In agreement with this finding, Qiu et al. reported that BMI at an early age was positively correlated with CVD [24]. Similarly, a previous study reported that obesity directly contributes to the development of cardiovascular risk factors. Obesity independently contributes to the onset of CVD and associated mortality, regardless of other cardiovascular risk factors [25]. This finding highlights the importance of BMI in cardiovascular risk stratification, indicating that individuals with a normal BMI may nevertheless be vulnerable to cardiac abnormalities in the setting of diabetes. Consequently, weight management and metabolic health remain important considerations for patients with diabetes, and regular cardiac monitoring should be considered regardless of BMI status. This underscores the need for comprehensive cardiovascular risk evaluation and preventive measures targeting both obese and non-obese diabetic populations. Furthermore, in addition to the evaluated risk factors for CVD in DM, numerous comorbidities may increase cardiovascular risk in diabetic individuals, including COVID-19, which can cause vascular endothelial damage and subsequently increase the risk of cardiovascular and cerebrovascular disease [26]. This significant issue requires further focused, prospective research.

This study has several limitations that should be considered. The retrospective design may have introduced selection bias that could not be controlled. Furthermore, because of the small sample size (n = 168) and retrospective data collection, there was a nonuniform distribution of age groups within the retrieved echocardiographic records. Patients aged ≤60 years constituted a larger proportion of the dataset, which may have resulted in sampling imbalance. Therefore, the crude associations observed in the univariate analysis may reflect this imbalance rather than a true increased risk among these age groups. This is further supported by the multivariate model, in which age did not remain statistically significant after adjustment, indicating that age was not an independent predictor of echocardiographic abnormalities.

Additionally, incomplete records may have limited the ability to establish causal relationships. The study was conducted in only two hospitals in the Jazan region; therefore, the generalizability of the findings to other regions of Saudi Arabia and other populations may be limited. Important clinical and demographic variables, such as medication history, lifestyle factors, comorbidities, and other associated factors, were not assessed and may have influenced the results and conclusions. The lack of longitudinal data also prevents the evaluation of changes in cardiac abnormalities over time and their clinical significance. Variability in assessments among different specialists was not accounted for, which may also have affected the findings. Another limitation is that the severity of mitral and tricuspid regurgitation was not considered in the recorded echocardiographic findings. In most examinations, mitral and tricuspid regurgitation occurred concurrently rather than as isolated findings. Without distinguishing physiological trace or mild regurgitation from clinically significant valvular disease, the overall prevalence of abnormalities may have been overestimated.

Moreover, the age criteria used in the inclusion and exclusion criteria may have been arbitrary, and residual confounding factors may remain. Given these limitations, the findings should be interpreted with caution. Future multicenter, prospective studies incorporating additional variables and longitudinal follow-up are needed to further evaluate cardiac abnormalities in patients with T2DM.

## Conclusions

This study revealed a significant frequency of echocardiographic abnormalities in patients with T2DM in the Jazan region, with mitral regurgitation, LVH, and tricuspid regurgitation identified as the most prevalent findings. While certain factors showed significant crude associations, age, gender, diabetes duration, and HbA1c values were not independently associated with the presence of echocardiographic abnormalities in the multivariate model. These results underscore the need for regular echocardiographic screening and comprehensive risk assessment in patients with T2DM, regardless of age or gender, to facilitate early identification and intervention. Future research should focus on prospective, multicenter studies incorporating a broader range of clinical factors and longitudinal follow-up to enhance understanding of the progression and outcomes of cardiac complications in this high-risk population.
